# Changes in pneumococcal carriage prevalence and factors associated with carriage in Norwegian children, four years after introduction of PCV13

**DOI:** 10.1186/s12879-019-4754-0

**Published:** 2020-01-10

**Authors:** A. Løvlie, D. F. Vestrheim, I. S. Aaberge, A. Steens

**Affiliations:** 1 Division for Infection Control and Environmental Health, Norwegian Institute of Public Health (NIPH), P.o.box 222 Skøyen, 0213 Oslo, Norway; 2 European Program for Intervention Epidemiology Training (EPIET), European Centre for Disease Prevention and Control (ECDC), Stockholm, Sweden

**Keywords:** Carriage, Pneumococcal conjugate vaccine

## Abstract

**Background:**

*Streptococcus pneumoniae* carriage is often asymptomatic but can cause invasive pneumococcal disease. Pneumococcal carriage is a prerequisite for disease, with children as main reservoir and transmitters. Childhood carriage can therefore be used to determine which serotypes circulate in the population and which may cause disease in the non-vaccinated population. In 2006, a pneumococcal conjugate vaccine (PCV7) was introduced into the Norwegian Childhood Immunisation Programme, which was replaced by the more valent PCV13 in 2011. We investigated changes in pneumococcal carriage prevalence 4 years after switching to PCV13 compared to three previous surveys, and analysed factors associated with carriage in children.

**Methods:**

We conducted a cross-sectional study in Norway, autumn 2015, among children attending day-care centres. We collected questionnaire data and nasopharyngeal swabs to identify pneumococcal serotypes. We compared the carriage prevalence in 2015 with surveys conducted in the same setting performed before widespread vaccination (2006; *n* = 610), 2 years after PCV7 introduction (2008; *n* = 600), and 2 years after switching to PCV13 (2013; *n* = 874). Using multilevel logistic regression we determined the association between pneumococcal carriage and previously associated factors.

**Results:**

In 2015, 896 children participated, with age ranging from 8 to 80 months. The overall carriage prevalence was 48/100 children [95%CI 44–53] in 2015, 38% [29–46] lower than in 2006 pre-PCV7, and 23% [12–32] lower than in 2013, 2 years after switching to PCV13. The PCV13 carriage prevalence was 2.8/100 children [1.9–4.2] in 2015. Increasing age (*p* < 0.001), recent antimicrobial use (odds ratio = 0.42 [0.21–0.57]) and being vaccinated (odds ratio = 0.37 [0.29–0.47]) were negatively associated with carriage.

**Conclusions:**

Our study showed a continued decrease in overall pneumococcal carriage, mainly fuelled by the decline in vaccine serotypes after vaccine introduction. Childhood vaccination with PCV13 should be continued to keep low PCV13 carriage, transmission and disease. Furthermore, the low prevalence of PCV13-type carriage in children endorse the choice of not recommending PCV13 in addition to the 23-valent pneumococcal polysaccharide vaccine to most medical risk groups in Norway, as little disease caused by these serotypes can be expected.

## Background

*Streptococcus pneumoniae* (pneumococcus) can cause non-invasive and invasive pneumococcal disease (IPD), including bacteraemia and meningitis [1, 2]. Pneumococcal nasopharyngeal carriage is a prerequisite for disease. Children are the main reservoir for pneumococci [2, 3] and globally the highest burden of IPD is in children and elderly people [2]. Before widespread vaccination with pneumococcal conjugate vaccines (PCV), the global number of deaths among under-fives caused by pneumococcal pneumonia was estimated to be 642,000 in 2005, a considerable proportion of the total number of 1,692,300 fatal cases [4]. The IPD incidence in high-income countries then ranged from 17.1 to 94.7/100000 child-years [5]. Before introduction of PCV in the Norwegian Childhood Immunisation Programme, pneumococci caused more than 1000 cases of IPD yearly in all age groups [6], and the incidence of IPD among the < 5 years olds was around 36/100,000 [7].

PCVs have a direct effect on the incidence of pneumococcal disease in vaccinated individuals. Furthermore, as PCVs also prevent vaccine type (VT) carriage, non-vaccinated individuals are indirectly protected by preventing further spread [8]. At least 97 pneumococcal serotypes have been identified [9], and available vaccines provide protection against a subset of these serotypes. In 2006, a vaccine protecting against seven serotypes (PCV7) was introduced into the Norwegian Childhood Immunisation Programme. The vaccine was replaced with the 13-valent vaccine (PCV13) in 2011, which protects against six additional serotypes. The vaccine is administered at three, five and twelve months of age. In 2015, the national uptake of PCV13 (three doses) in two-year-olds was 95% [10].

After introduction of PCV7, a rapid decrease in the incidence of PCV7-type IPD was observed in all age groups in several sites, including Norway [6, 11]. However, the incidence of IPD caused by non-vaccine serotypes (NVT) increased [6], particularly for serotype 19A [1, 6, 11–13]. The overall prevalence of carriage among children remained stable, due to a decrease in PCV7-type carriage and an increase in non-PCV7 type carriage (called serotype replacement) [6, 12, 14–17].

Factors found to be positively associated with pneumococcal carriage before and after PCV7 introduction included younger age [2, 18–20], attendance in day-care centres (DCC) [18, 21], larger family size [2, 18, 19, 21], history of recent respiratory tract infection (RTI) [21], and passive smoking [18], whereas recent exposure to antimicrobials has been found to be negatively associated with carriage [19]. It is unknown whether these factors are still of relevance now that PCV13 has been used on a larger scale.

In light of the changes in the Childhood Immunisation Programme, it is important to monitor the prevalence of carriage and distribution of VT and NVT carriage in children over time to be able to inform vaccine policy, i.e. to make validated choices of which vaccines to include in the vaccination programs, both for children and other risk groups. This is particularly important given the nature of the pneumococcus with highest carriage prevalence among young children [3, 18, 19], resulting in children therefore being the main transmitters in the population [2]. Childhood carriage can therefore be used to determine which serotypes circulate in the population and which may cause disease in the vulnerable population. Studies on carriage after introduction of PCV13 have already shown changes in PCV13-type and NVT carriage, though most have been conducted within the first 2 years of the implementation of the vaccine [12, 17, 22]. Using integrated data from surveys performed in 2006, 2011, 2013, and 2015, we aimed to describe changes in the pneumococcal carriage prevalence 4 years after switching from PCV7 to PCV13 in the Childhood Immunisation Programme in Norway, and to determine which factors were associated with pneumococcal carriage in children attending DCC.

## Methods

**Fig. 1:**
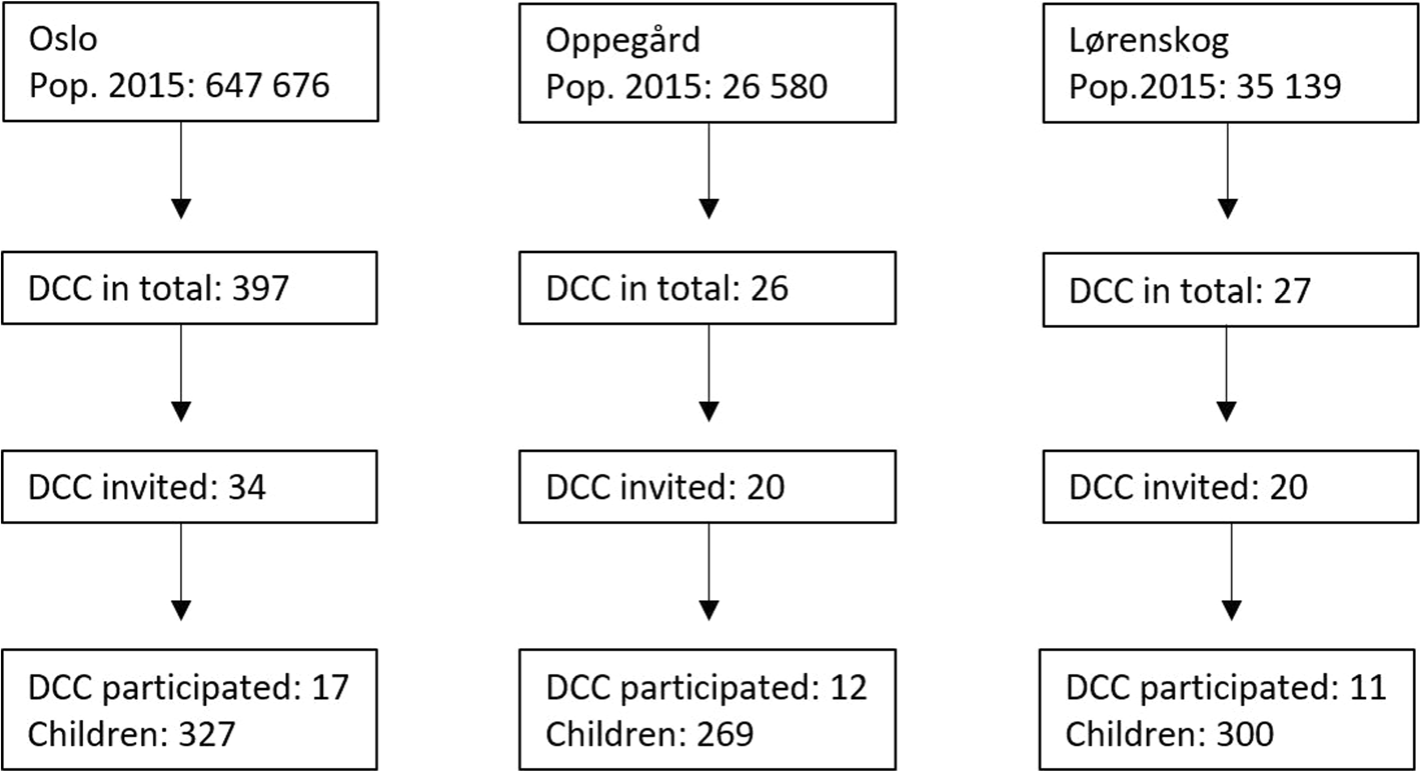
Flow chart of the recruitment of day care centres (DCC) in the 2015 survey. Pop.2015 = the population living in the municipality at January 1th, 2015 as published by Statistics Norway

We performed a cross-sectional carriage study in Norway among children in DCC in 2015, 4 years after switching to PCV13. Data were collected from September to November. We invited a convenience sample of DCC in two municipalities neighbouring Oslo and a random sample of DCC in Oslo, see Fig. 1. From each DCC, all children were invited to participate; no exclusion criteria. Based on the previous surveys we performed a sample size calculation by taking into account the design effect, i.e. 1.6, and the average cluster size. We estimated that we needed at least 800 children to detect a change in prevalence of 10%. Note that 90% of children aged 1–6 year attend DCC in Norway [23].

Study nurses were present in DCC for up to two full days (depending on the size of the DCC) for data collection. Parents had received information about the study beforehand. All parents or guardians dropping off or picking up their children at the DCC were invited to participate and written informed consent was obtained from the parents or guardians before participation. Parents or guardians filled out a questionnaire on demographics and the following factors: time being breastfed, cohabitating with siblings aged < 6 years, vaccine history, passive smoking, history of a RTI during the last 3 months (yes/no; called recent RTI in the text) and use of antimicrobials during the last 3 months (yes/no; called recent antimicrobial use in the text). See the Additional file 1: for an English translation of the questionnaire. Data on the size of the DCC were collected at the day of sampling (reported by the staff). Vaccine history was defined as being vaccinated with at least one dose of any PCV (yes/no), independent of vaccine type.

From all participating children we obtained a nasopharyngeal swab. The swabs were stored and transported in a medium containing skim milk powder, tryptone soy broth, glucose and glycerol in distilled water (STGG) and were stored at − 70 °C within 4 h after sampling. Within 1 month after sampling, the samples were processed further: 200 μl of STGG was added to 3 ml enrichment broth and 20 μl STGG was plated on gentamycin-blood-agar plates. The broths and plates were incubated overnight at 35 °C with 5% CO_2_. Pneumococci were identified using a latex agglutination test (Pneumotest-Latex kit; Statens Serum Institut) from the incubated broths. Confirmation and serotyping was done by Quellung reaction using serotype-specific sera. All morphological different colonies were serotyped. If the latex agglutination test indicated presence of more serotypes, up to 16 colonies were isolated in the attempt to identify all serotypes. More details on data collection procedures, laboratory specimen sampling, transport and analyses can be found in Steens et al and Vestrheim et al [22, 24, 25]. Serotypes were categorised as PVC7 (serotypes 4, 6B, 9 V, 14, 18C, 19F, 23F), serotypes that are in PCV13 but not in PCV7 (PCV13–7; serotypes 1, 3, 5, 6A, 7F, 19A) and NVT (all other serotypes).

We compared the results of the 2015 survey to the 2006, 2008, and 2013 surveys, all with a similar design, conducted during the same season and in the same population before widespread vaccination of children (2006), 2 years after introducing PCV7 (2008) [14] and 2 years after switching to PCV13 (2013) [22]. See Additional file 1: Table S1 for survey sizes and characteristics of the study participants in the different surveys.

The authors assert that all procedures contributing to this work comply with the ethical standards of the relevant national and institutional committees on human experimentation (approved by the Regional Committee for Medical Research Ethics, South-Eastern Norway; 2014/2046) and with the Helsinki Declaration of 1975, as revised in 2008.

### Statistical analyses

All analyses were done correcting for the cluster design with DCC as sampling unit, i.e. the dependencies in the data. We determined percentages for the demographical data and calculated carriage prevalence by serotype category per 100 children, including the 95% confidence interval [95%CI], using the survey command in Stata 15. We performed univariable multilevel logistic regression analysis (melogit command in stata) to determine differences between sub-categories. DCC was entered as random intercept to correct for the cluster-sampling design. To test significance for linear and binary variables we used single parameters Wald-tests, while significance for categorical variables with more than two categories was tested using multiple parameter Wald-tests.

To estimate the change in prevalence since PCV7 introduction and to determine the recent change in PCV13 carriage, we estimated 1-carriage prevalence ratios*100% for 2015:2006 and 2015:2013, respectively, using Poisson regression within the survey command.

We performed univariable and multivariable multilevel logistic regression analysis, estimating odds ratios (OR) to determine the association between pneumococcal carriage and previously associated factors. We included the following variables in the multivariable model: age in months, cohabitating with siblings < 6 months, breastfed < 2 months, passive smoking, vaccine history, use of antimicrobials last 3 months and size of day care centre. We used either all serotype, PCV13 or NVT carriage as outcome. We excluded the variable on recent RTI from the multivariable model, as this was strongly associated with recent antimicrobial use. Similarly, because of the strong association between vaccine history and study year due to the changes in the immunisation program, we did not include study year in the multivariable analysis. We combined all individuals from the four surveys in the analysis (2006, 2008, 2013 and 2015). .

All analyses were done in Stata 15. Our level of statistical significance was defined as α = 0.05.

## Results

### The 2015 sample, four years after switching from PCV7 to PCV13

In 2015, 896 children from 40 DCC participated in the study, reflecting a median response rate of 45%(see Additional file 1: Table S1). The age of participants ranged from eight to 80 months, with a median of 44 months. Fifty-two percent (95%CI [49–55%]) were boys. Ninety-nine percent (95%CI [98–100%]) reported to have been vaccinated; five children were unvaccinated (Table 1).

**Table 1:** Characteristics of the study participants in 2015, 4 years after switching to PCV13

Variable	Number of children (percentage [95%CI])
*Demographics*	
Age groups	
< 24 months	139 (16% [13–19%])
24–35 months	168 (19% [16–22%])
36–47 months	195 (22% [19–24%])
48–59 months	208 (23% [20–27%])
> = 60 months	186 (21% [18–23%])
Number of boys	464 (52% [49–55%])
*Factors previously associated with carriage*	
Cohabitating with siblings < 6 years old	436 (49% [45–53%])
Being breastfed < 2 months	48 (6% [4–8%])
Passive smoking	39 (4% [3–6%])
Vaccine history	881 (99% [98–100%])
Vaccinated by type	
PCV7 only	58 (7% [5–9%])
PCV7 + PCV13	173 (20% [17–23%])
PCV13 only	650 (73% [70–77%])
Having had respiratory tract infection during the past 3 months	75 (8% [6–11%])
Having used antimicrobials during the past 3 months	69 (8% [6–9%])
Number of children per day-care centre (DCC), by category	
10–29 children (9 DCCs)	122 (14% [67–26%])
30–49 children (11 DCCs)	253 (28% [15–46%])
50–69 children (11 DCCs)	277 (31% [17–49%])
70–89 children (8 DCCs)	214 (24% [11–43%])
90+ children (1 DCC)	30 (3% [0.4–22%])

The overall carriage prevalence in 2015 was 48.1/100 children [43.7–52.5] (Table 2). The highest prevalence was observed among the age group < 24 months (60.4/100 children), and the prevalence decreased with increasing age (*p* = 0.008). The overall carriage prevalence of PCV13 serotypes was 2.8/100 children [1.9–4.2], with 1.3/100 carrying PCV7-serotypes and 1.5/100 carrying PCV13–7 serotypes. The overall prevalence of NVT carriage was 46.0/100 children [41.5–50.5], with 60.4/100 among the < 24 months old. The overall and PCV13–7 carriage prevalence were highest among children vaccinated with PCV13 only (*p* < 0.001). However, it should be noted that children vaccinated with PCV13 were inherently younger than those vaccinated with PCV7. The carriage prevalence was slightly lower, but not statistically significant (*p* = 0.220), in children with reported recent antimicrobial use (40.6/100 [30.9–51.1]) than in those with no recent antimicrobial use (48.5/100 [44.0–53.1]). We did not find differences in carriage prevalence by any of the other investigated factors (see Additional file 1: Table S2).

**Table 2:** Carriage prevalence per 100 children, overall and by vaccine-type and non-vaccine type, in 2015

Variable	Number of carriers per subgroup	Prevalence carriage, per 100 children [95%CI]	Prevalence PCV7^a^ carriage number; prevalence per 100 children [95%CI]	Prevalence PCV13–7^b^ carriage number; prevalence per 100 children [95%CI]	Prevalence NVT^c^ carriage number; prevalence per 100 children [95%CI]
Total	431	48.1 [43.7–52.5]	12; 1.3 [0.7–2.4]	13;1.5 [0.8–2.6]	412; 46.0 [41.5–50.5]
Age in months	431 included				
< 24 months	84	60.4 [52.2–68.1]	0; 0 [NA]	1; 0.72 [0.09–5.4]	84; 60.4 [52.2–68.1]
24–35 months	94	56.0 [48.1–63.6]	2; 1.2 [0.28–5.0]	1; 0.60 [0.08–4.4]	91; 54.2 [46.0–62.1]
36–47 months	105	53.9 [46.0–61.5]	4; 2.0 [0.61–6.7]	6; 3.0 [1.5–6.1]	98;50.3 [42.8–57.7]
48–59 months	89	42.8 [35.5–50.5]	2; 0.96 [0.25–3.6]	5; 2.4 [0.85–6.6]	84; 40.4 [33.0–48.3]
> = 60 months	59	31.7 [24.6–39.8]	4; 2.2 [0.83–5.5]	0; 0 [NA]	55; 29.6 [22.8–37.4]
Vaccinated by type	428 included				
PCV7	20	34.5 [22.9–48.3]	1; 1.7 [0.22–12.1]	0; 0 [NA]	19; 32.8 [21.1–47.0]
PCV7+13	52	30.1 [23.0–38.3]	2; 1.2 [0.28–4.7]	1; 0.58 [0.08–4.1]	49; 28.3 [21.7–36.9]
PCV13	354	54.5 [50.0–58.9]	8; 1.2 [0.54–2.8]	12; 1.9 [0.98–3.5]	340; 52.3 [47.7–56.9]
Unvaccinated	2	40.0 [11.1–78.0]	1; 20.0 [6.7–46.7]	0; 0 [NA]	1; 20.0 [6.7–46.7]
Having used antimicrobials the past 3 months	428 included				
Yes	28	40.6 [30.9–51.1]	0; 0 [NA]	0; 0 [NA]	28; 40.6 [30.9–51.1]
No	400	48.5 [44.0–53.1]	12; 1.5 [0.80–2.6]	12; 1.5 [0.78–2.7]	382; 46.4 [41.7–51.1]

^a^carriage of serotypes covered by the 7-valent pneumococcal conjugate vaccine

^b^carriage of serotypes covered by the 13-valent pneumococcal conjugate vaccine but not by the 7-valent vaccine

^c^carriage of serotypes not covered by the 13-valent pneumococcal conjugate vaccine

*NA* not applicable (the 95% could not be determined because  no children in this category carried these types)

### Comparing the 2015 survey with the surveys performed in 2006, 2008 and 2013

**Fig. 2:**
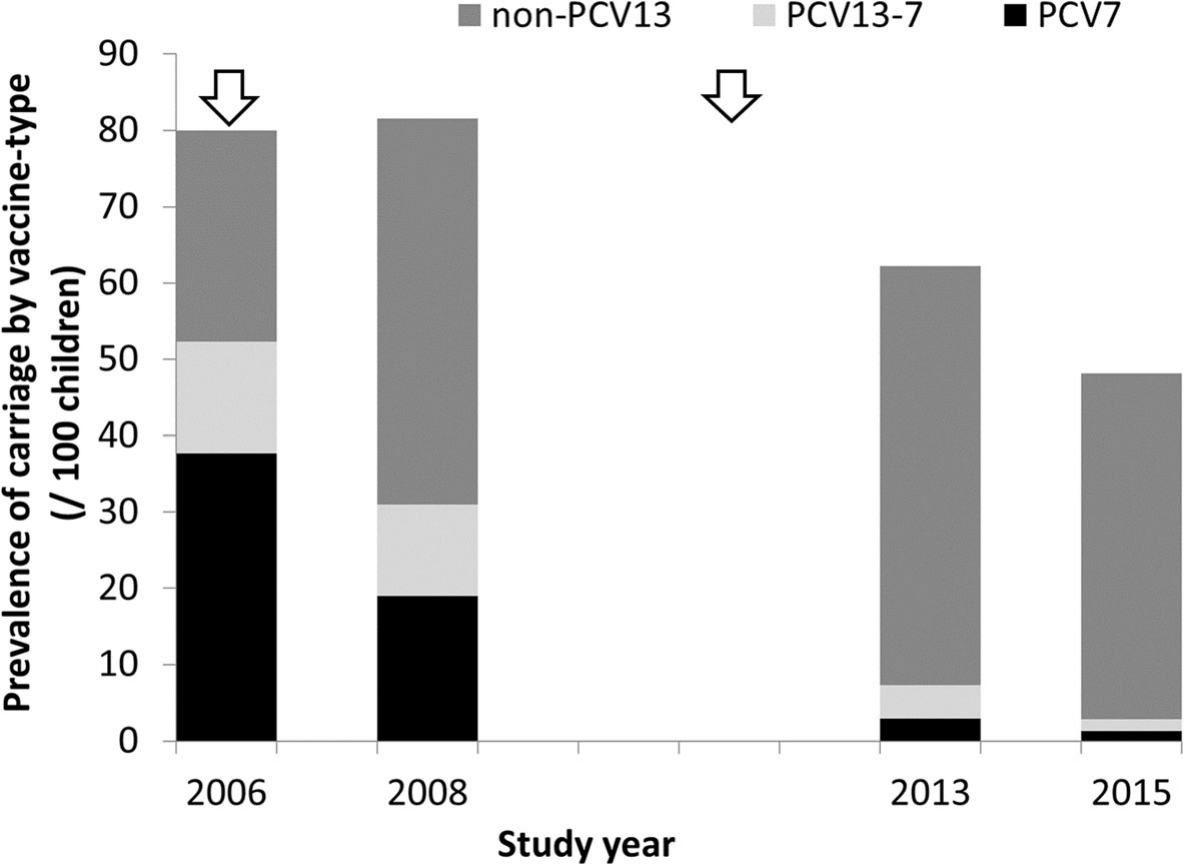
The prevalence of carriage by vaccine-type per study year, expressed per 100 children. The arrows indicate the timing of vaccine introduction (PCV7 in 2006, PCV13 in 2011). PCV7 = carriage of serotypes covered by the 7-valent pneumococcal conjugate vaccine; PCV13–7 = carriage of serotypes covered by the 13-valent pneumococcal conjugate vaccine but not by the 7-valent vaccine: NVT = carriage of serotypes not covered by the 13-valent pneumococcal conjugate vaccine

The combined dataset of the four surveys included 2980 participants. For detailed descriptive analyses of the first three surveys, see Steens et al and Vestrheim et al [22, 24]. In summary, 610 children participated in 2006, 600 in 2008 and 874 in 2013. The age and sex distributions did not differ between the study years; see Additional file 1: Table S1. In 2015, the carriage prevalence was 38% [29–46] lower than in 2006 pre-PCV7, and 23% [12–32] lower than in 2013, 2 years after switching to PCV13 (Fig. 2). The decrease in carriage compared to the 2013 survey was caused by a significant decrease of 62% [36–77] in PCV13 carriage prevalence and of 18% [7–27] in NVT carriage prevalence. See Additional file 1: Figure S1 for individual serotype data.

### Factors associated with carriage

In the univariable regression analyses, the variables study year, age group, vaccine history, recent RTI and recent antimicrobial use were significantly associated with any pneumococcal carriage (Table 3). In the multivariable regression analysis (excluding study year and recent RTI; see methods section), vaccine history (OR = 0.37 [0.29–0.47]), recent antimicrobial use (OR = 0.42 [0.32–0.57]) and age (Wald-test *p* < 0.001) remained negatively associated with carriage. If we used PCV13 carriage as outcome, the negative association with vaccine history was even stronger (OR 0.13 [0.09–0.18]). The multivariable analysis between NVT carriage and vaccine history showed a positive association (OR 1.7 [1.3–2.1]). From the first survey in 2006 to the last survey in 2015, the percentage of children being vaccinated with at least one dose of any PCV increased from 3% [2–6] in 2006, to 40% [35–44] in 2008, to 99% [98–99] in 2013 and 99% [98–100] in 2015. Recent antimicrobial use did not differ significantly over time; in 2006, 9% [6–13] reported recent use of antimicrobials, compared to 5% [4–8] in 2008, 10% [8–12] in 2013 and 8% [6–9] in 2015.

**Table 3:** Univariable and multivariable multilevel logistic regression analyses between any pneumococcal carriage and factors previously associated with carriage; data of the 2006, 2008, 2013 and 2015 surveys were included

Factors previously associated with carriage	Univariable: Odds Ratio (OR) [95%CI]	*p*-value^b^	Multivariable: Odds Ratio (OR) [95%CI]^c^	*p*-value^b^
Year of the study sample		< 0.001	–	–
2006	3.9 [2.9–5.1]			
2008	4.5 [3.4–6.0]			
2013	1.8 [1.5–2.2]			
2015	1			
Age in months		< 0.001		< 0.001
**<** 24	1		1	
24–35	0.99 [0.74–1.3]		0.94 [0.70–1.3]	
36–47	0.74 [0.56–0.98]		0.61 [0.46–0.82]	
48–59	0.54 [0.41–0.70]		0.41 [0.31–0.54]	
> = 60	0.39 [0.29–0.52]		0.31 [0.22–0.40]	
Cohabiting with siblings < 6 years old		0.204		0.402
No	1		1	
Yes	1.1 [0.95–1.3]		1.1 [0.91–1.3]	
Breastfed < 2 months		0.567		0.665
No	1		1	
Yes	1.1 [0.78–1.6]		1.1 [0.76–1.5]	
Passive smoking		0.685		0.895
No	1		1	
Yes	1.1 [0.81–1.4]		0.98 [0.73–1.3]	
Vaccinated		< 0.001	–	–
PCV7	1			
PCV7 + 13	0.60 [0.45–0.80]			
PCV13	0.98 [0.79–1.2]			
Unvaccinated	1.9 [1.5–2.4]			
Vaccine history^d^		< 0.001		< 0.001
No	1		1	
Yes	0.50 [0.39–0.64]		0.37 [0.29–0.47]	
RTI^a^ last 3 months		< 0.001	–	–
No	1			
Yes	0.58 [0.44–0.76]			
Use of antimicrobials last 3 months		< 0.001		< 0.001
No	1		1	
Yes	0.48 [0.37–0.64]		0.42 [0.32–0.57]	
Size of day care centre		0.1905		0.7132
10–29 children	1		1	
30–49 children	1.3 [0.79–2.0]		1.2 [0.80–1.7]	
50–69 children	1.3 [0.81–1.9]		1.1 [0.80–1.6]	
70–89 children	1.6 [0.98–2.4]		1.2 [0.82–1.7]	
90+ children	2.0 [1.1–3.5]		1.4 [0.89–2.2]	

^a^*RTI* Respiratory tract infection

^b^Significance for linear and binary variables was tested using single parameters Wald-tests; significance for categorical variables with more than two categories was tested using multiple parameter Wald-tests

^c^In the multivariable model we included the following variables: age in months, cohabitating with siblings < 6 months, breastfed < 2 months, passive smoking, vaccine history, use of antimicrobials last 3 months and size of day care centre

^d^Vaccine history was defined as being vaccinated with any PCV (yes/no; at least one dose), independent of vaccine type

## Discussion

In this study, we identified a continued decrease in overall pneumococcal carriage and carriage of PCV13 pneumococci 4 years after switching from PCV7 to PCV13 in the Norwegian Childhood Immunisation Programme. While carriage of NVTs had increased 2 years after introduction of PCV7 (2008) and 2 years after the switch to PCV13 (2013) [14, 22], the NVT prevalence had not increased further in 2015, but had decreased compared to 2013. Furthermore, we found that recent antimicrobial use, vaccine history and older age were negatively associated with carriage.

A reduction in overall carriage prevalence after the introduction of PCV7 and/or PCV13 has been observed before [12, 15, 16, 21, 26], though in several other settings the overall carriage prevalence remained the same [17, 27–29]. The decrease in carriage has mainly been driven by the reduction in VT carriage [12, 15, 16, 21, 26]. While many studies showed only some concurrent increase in NVT prevalence, in several studies the decrease in VT and increase in NVT carriage was of similar size leading to near-complete replacement [17, 27–29]. The size of the changes varied between studies [12, 15–17, 21, 30], which may be related to the study population (e.g. hospitalised [30] versus healthy children [17]), vaccination coverage [15, 16, 21] or the pre-vaccination serotype distribution.

Even though the increase in carriage of NVT found by others [27, 31–34] corresponds well with what we have observed in Norway after PCV7 introduction [14], we did not observe near-complete replacement after the switch to PCV13 [22]. In our 2015 study, we documented a statistically significant decrease in carriage of NVT compared to 2013, which has not been described in other countries. This decrease might be due to vacated niches in the nasopharynx that may have become occupied by other bacteria than pneumococci or due to secular trends, i.e., variation over time, not related to vaccination. Replacement with other bacteria was indicated by the long-term follow up of a randomised controlled trial in Dutch children vaccinated with PCV7, where an increase in carriage of *Haemophilus influenzae* and *Staphylococcus aureus* together with a decrease in pneumococcal carriage was observed [35]. Still, we cannot exclude methodological reasons as we had changed the transport medium from enrichment broth in the three earlier surveys to STGG in 2015, though an in vitro and in vivo comparison of the methods indicated no statistical difference between them [25].

Several studies looked already into factors associated with carriage, including age [2, 18–20], family size [2, 17, 18, 20], DCC attendance [18, 21], passive smoking [18], recent RTI [21], and recent antimicrobial use [19]. We found that vaccine history, older age and recent antimicrobial use were negatively associated with carriage. Young age is an established risk factor for carriage [16, 18, 36, 37], and can be explained by the maturation of both serotype-specific and non-specific immune responses to pneumococci as children grow older [2]. Recent antimicrobial use was also found to be negatively associated with carriage by several other studies [16, 36, 37], which is plausible as the use of antimicrobials reduces many microbes in the nasopharynx. We found that recent RTI was negatively associated with carriage, but participants recently using antimicrobials and those reporting recent RTI overlapped. This led us to believe that the recent antimicrobial use caused the negative association with carriage, as it has been previously shown that pneumococcal carriage increases during RTIs and decreases after treatment with antimicrobials [38, 39].

While cohabitating with multiple siblings, short duration of breastfeeding, passive smoking and the size of DCC have been associated with pneumococcal carriage before, we did not find such associations. This may partly be explained by the power of our study and the composition of our study population. Breastfeeding for fewer than 2 months is uncommon in Norway, where > 90% of children are still breastfed at 3 months of age [40]. Living in a larger family, cohabitating with young siblings and attending DCC are all related to the increased risk of transmission in crowded surroundings. As we only included children attending DCCs, all children were exposed to crowded settings, thereby making it more difficult to find differences.

Strengths of our study are the large size and the repetition of the same study design in the same population during the same season. However, our study also has limitations. We only recruited participants from DCC in Oslo and the surrounding region, which may limit the representativeness for the entire Norwegian child population. However, this can probably be disregarded as the vast majority (90%) of children in the included age groups attend DCC in Norway [23]. Furthermore, we collected self-reported data, such as vaccine history, recent RTI and antimicrobial use, without subsequent verification. Data from the Norwegian Prescription Registry show similar percentages of antimicrobial use in this population [41], and data from the Norwegian Immunisation Registry (SYSVAK) show a similar coverage for PCV-vaccination [10], indicating that the reported data are likely to be reliable. Passive smoking may have been underestimated, as there is a social stigma to smoking indoors in Norway, especially in the presence of children. Another limitation is that the questionnaire was only available in Norwegian, limiting the participation of parents who did not speak Norwegian. However, the number of non-Norwegian parents was very small and an interview in English was offered as an alternative to them. Occurrence of IPD and pneumococcal carriage follows a winter-seasonal pattern in temperate climates, probably related to an increased carriage of pneumococci and an increased susceptibility to develop IPD [42]. However, since our data was collected at the same time all four study years, the difference in carriage prevalence is unlikely to be related to seasonal differences in our data.

## Conclusion

Overall, the results of this study showed a continued decrease in overall *S. pneumoniae* carriage, mainly fuelled by the dramatic decrease of PCV13 carriage after vaccine introduction. Vaccine history, older age and use of antimicrobials within the last 3 months were negatively associated with carriage. Childhood vaccination with PCV13 should be continued to keep low PCV13 carriage, transmission and disease. Furthermore, the low prevalence of PCV13-type carriage in children endorses the choice of not recommending PCV13 as main vaccine to medical risk groups in Norway, as little disease caused by PCV13 serotypes can be expected [43].

## Supplementary information

**Additional file 1:** The additional file includes the following material: An English translation of the questionnaire used in the study; **Table S1.** Characteristics of the surveys and study participants in 2006, 2008, 2013 and 2015; **Table S2.** Carriage prevalence per 100 children shown for the variables previously associated with carriage that have not been presented in Table 2 of the main article; **Figure S1.** Carriage prevalence per 100 children shown per serotype, separately for PCV7-type carriage, carriage of serotypes that are included in PCV13 but not in PCV7 (PCV13–7) and non-vaccine type (NVT) carriage.

## Data Availability

The datasets used and analysed during the current study are available from the corresponding author on reasonable request.

## Abbreviations

95%CI: 95% confidence interval
DCC: Day-care centres
IPD: Invasive pneumococcal disease
NVT: Non-vaccine serotypes
OR: Odds ratio
PCV7/PCV13: Seven/thirteen valent pneumococcal conjugate vaccine
PCV13-7: serotypes that are in PCV13 but not in PCV7
RTI: Respiratory tract infection
STGG: Medium containing skim milk powder, tryptone soy broth, glucose and glycerol in distilled water
VT: Vaccine serotype
