# Supplementary material for: Changes in pneumococcal carriage prevalence and factors associated with carriage in Norwegian children, four years after introduction of PCV13
Source: BMC Infect Dis. 2020 Jan 10;20:29. doi: 10.1186/s12879-019-4754-0 (PMC6954625; doi:10.1186/s12879-019-4754-0)
Supplement: Supplementary file 1 — Additional file 1: The additional file includes the following material: An English translation of the questionnaire used in the study; Table S1. Characteristics of the surveys and study participants in 2006, 2008, 2013 and 2015; Table S2. Carriage prevalence per 100 children shown for the variables previously associated with carriage that have not been presented in Table 2 of the main article; Figure S1. Carriage prevalence per 100 children shown per serotype, separately for PCV7-type carriage, carriage of serotypes that are included in PCV13 but not in PCV7 (PCV13–7) and non-vaccine type (NVT) carriage. [file 12879_2019_4754_MOESM1_ESM.docx]

Supplementary material belong to **Changes in pneumococcal carriage prevalence and factors associated with carriage in Norwegian children, 4 years after introduction of PCV13**, by: A. Løvlie, D.F. Vestrheim, I.S. Aaberge and A. Steens

**English translation of the questions asked in the questionnaire**

Date of birth of the child

Initials

Study ID of siblings in the study

Name of the day care centre

Postal code

Sex

How many people live in the same household as the child?

Age of each child living in the same household as the participating child

When did the child start in day care (this day care centre or another)?

Was the child breastfed / did the child receive breast milk? Yes / no, if yes, for how many months?

Does someone smoke in the household of the child? [note that information was provided on that we meant smoking inside the house]

Did the child have an infection/inflammation during the last 3 months? No / yes: ear infection, throat infection, pneumonia, different disease (which)

Did the child receive any antibiotics for the infection during the last 3 months (e.g. penicillin)?

Did the child receive all vaccines of the childhood vaccination programme? Yes / no: if no, which vaccines are given (age or name of the vaccine)

**Table S1. Characteristics of the surveys and study participants in 2006, 2008, 2013 and 2015**

|  | **2006** | **2008** | **2013** | **2015** |
| --- | --- | --- | --- | --- |
| **Municipalities** | Lørenskog and Oppegård | Lørenskog and Oppegård | Lørenskog, Oppegård and Oslo | Lørenskog, Oppegård and Oslo |
| **Sampling period** | September 13 to November 7 | September 3 to October 28 | September 9 toNovember 7 | September 7 to November 13 |
| **Number off day-care centres (number in Lørenskog+Oppegård+Oslo)** | 29 (11+18+0) | 27 (10+15+0) | 40 (12+17+11) | 40 (11+12+17) |
| **Median response rate within day-care centres (%)** | 40 | 43 | 42 | 45 |
| **Number of boys (%)** | 326 (53) | 314 (52) | 445 (51) | 464 (52) |
| **Median age in months (range)** | 45 (5-69) | 42 (6-69) | 46 (10-77) | 44 (8-80) |
| **Number of participating children** | 610 | 600 | 874 | 896 |
| **Number of pneumococcal isolates** | 538 | 562 | 583 | 463 |

| **Table S2. Carriage prevalence per 100 children shown for the variables previously associated with carriage that have not been presented in Table 2 of the main article. Presented as any serotype carriage and by vaccine-type/non vaccine type-carriage, in 2015.** Overall, 896 children were included in the survey, of whom 431 were carriers. If data on certain variables was missing for some children, the number of carriers that could be included in the subgroup analysis is presented in the second column. | | | | | |
| --- | --- | --- | --- | --- | --- |
| **Variable** | Number of carriers per subgroup | **Prevalence of any serotype carriage** number; prevalence per 100 children [95%CI] | **Prevalence of PCV7 carriage**  number; prevalence per 100 children [95%CI] | **Prevalence of PCV13-7 carriage** number; prevalence per 100 children [95%CI] | **Prevalence of NVT carriage** number; prevalence per 100 children [95%CI] |
| **Total** | **431** | **48.1 [43.7-52.5]** | **12; 1.3 [0.73-2.4]** | **13;1.5 [0.81-2.6]** | **412; 46.0 [41.5-50.5]** |
| **Sex** |  |  |  |  |  |
| Male  Female | 228  203 | 49.1 [43.5-54.8]  47.0 [41.0-53.1] | 4;0.86 [0.31-2.3]  8;1.9 [0.83-4.1] | 5;1.1 [0.38-3.0]  8;1.9 [0.99-3.5] | 220; 47.4 [41.8-53.1]  192; 44.4 [3.8-50.9] |
| **Cohabiting with siblings <6 years old**  Yes  No | 212  219 | 48.6 [43.7-53.6]  47.8 [42.0-53.7] | 4; 0.92 [0.28-3.0]  8; 1.7 [0.85-3.6] | 5; 1.2 [0.50-2.6]  8; 1.7 [0.86-3.5] | 205; 47.0 [41.9-52.2]  207; 45.2 [39.5-51.1] |
| **Being breastfed <2 months**  Yes  No | **396 included**  22  374 | 45.8 [33.5-58.7]  48.7 [44.5-53.0] | 2; 4.17 [1.0-15.8]  9; 1.2 [0.55-2.5] | 0; 0 [NA]  12; 1.6 [0.83-3.0] | 20; 41.7 [28.8-55.8]  359; 46.7 [42.4-51.2] |
| **Passive smoking**  Yes  No | **431 included**  19  412 | 48.7 [32.3-65.5]  48.1 [43.7-52.6] | 1; 2.6 [0.33-17.2]  11; 1.3 [0.67-2.4] | 1; 2.6 [0.40-14.8]  12; 1.4 [0.75-2.6] | 17; 43.6 [27.3-61.4]  395; 46.1 [41.6-50.7] |
| **Having had an RTI* during the past 3 months**  Yes  No | **430 included**  31  399 | 41.3 [31.1-52.4]  48.7 [44.1-53.4] | 1; 1.3 [0.18-9.4]  11; 1.3 [0.71-2.5] | 1; 1.3 [0.17-9.9]  12; 1.5 [0.78-2.7] | 29; 38.7 [28.1-50.4]  382; 46.6 [41.7-51.6] |
| **Size of day care centre**  10-29 children  30-49 children  50-69 children  70-89 children  90- children ** | 49  130  128  108  16 | 40.1 [29.8-51.5]  51.4 [43.3-59.4]  46.2 [38.3-54.4]  50.5 [41.7-59.2]  53.3 [53.3-53.3] | 2; 1.6 [0.47-5-6]  4; 1.6 [0.56-4.4]  4; 1.4 [0.45-4.5]  2; 0.9 [0.29-2.9]  0; 0 [NA]*** | 1; 0.82 [0.12-5.3]  6; 2.4 [0.85-6.5]  2; 0.72 [0.21-2.4]  4; 1.9 [0.98-3.5]  0; 0 [NA]*** | 46; 37.7 [27.7-48.9]  123; 48.6 [40.1-57.2]  124; 44.8 [37.0-52..8]  103; 48.1 [38.3-58.1]  16; 53.3 [53.3-53.3] |

* RTI= respiratory tract infection
** Only 1 DCC in the category
*** NA=not applicable as no children in this category carried these types


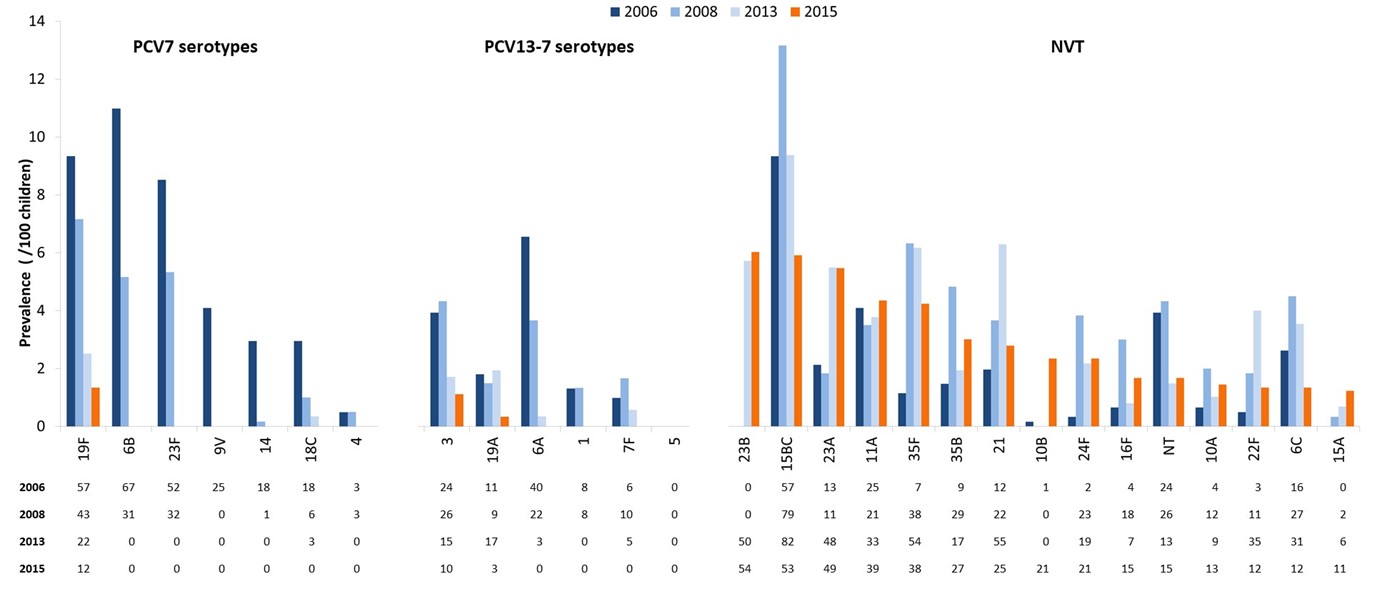
**Figure S1. Carriage prevalence per 100 children shown per serotype, separately for PCV7-type carriage, carriage of serotypes that are included in PCV13 but not in PCV7 (PCV13-7) and non-vaccine type (NVT) carriage.** The number of isolates per serotypes per survey are written under the serotype. Note that, for the non-vaccine serotypes, only those serotypes are presented that had a prevalence of >1/100 children in 2015. The numbers of isolates that are not presented in the figure are 32 for 2006, 52 for 2008, 59 for 2013 and 33 for 2015. The 2015 results are presented in orange; the 2006,2008 and 2013 studies in blue (see legend).
